# Novel approaches to identify and characterize young key population networks in southern Africa

**DOI:** 10.1002/jia2.25735

**Published:** 2021-06-30

**Authors:** Berry D Nibogora

**Affiliations:** ^1^ Independent consultant – Human Rights HIV Policies and Key Populations Johannesburg South Africa

**Keywords:** young key populations, HIV, SRHR, policy advocacy, youth empowerment, leadership, visibility

Key populations have always been disproportionately affected by the global HIV epidemic, and their relative risk continues to increase. In 2019, key populations (namely, men who have sex with men (MSM), sex workers of all genders, transgender individuals, prisoners and people who inject drugs) and their sexual partners accounted for 62% of new infections worldwide [1]. Their ability to keep themselves and others healthy and safe from HIV is compromised by multiple intersecting forms of inequality and stigma and discrimination, creating barriers at the individual, interpersonal, community and societal levels [1]. Young people who are members of one or more of these key populations face further challenges due to their life inexperience, lack of awareness or understanding of HIV risk factors and other age‐related factors, such as conservative family and community attitudes that deny young peoples’ ability to make decisions for themselves and unfettered access to HIV services.

Finding new ways to reach and support key populations and their partners is critical for sustained progress in HIV responses. A recent innovative approach involved engagement of young advocates from key populations to identify policy priorities and effectively engage representatives of government entities for policy change, as well as better inclusion of young key populations in the HIV and sexual and reproductive health and rights (SRHR) policy revision processes. This approach was tested in the programme that African Men for Sexual Health and Rights (AMSHeR) and two partners, the United Nations Development Programme (UNDP) and the Health Economics, AIDS and Research Department (HEARD) of the University of KwaZulu‐Natal, implemented from 2017 to 2020 in collaboration with civil society organizations, governments and the Southern Africa Development Community (full proposal available upon request). The approach used people in the communities as programme implementing officers, supported by a regional team to develop and implement empowerment and engagement strategies. It resulted in the emergence of a vibrant young key population leadership in Zimbabwe.

A key finding across all five countries – Angola, Madagascar, Mozambique, Zambia and Zimbabwe – was that direct engagement with and meaningful participation of African key population groups who are the end users of these interventions is needed in order to understand the factors that motivate uptake and adherence. The same approach also helps implementers tailor interventions to populations’ unique needs while working towards achieving universal coverage of HIV and SRHR services [2, 3]. Although programmes varied by the context in terms of implementation processes and outcomes, results observed in Zimbabwe provide an indicative example of approaches that offer promise in a variety of settings. The Zimbabwean case was selected based on the nature of the impact of the programme: change in SRHR policies.

## 1 Lessons learnt: an inclusive and participatory process allows for a new generation of leaders with bolder yet realistic priorities in Zimbabwe

In Zimbabwe, the programme used a model with a first step being the organization of in‐person dialogues that included a range of diverse young key populations (young gay men and other MSM, young sex workers of all genders, young drug users and young trans‐diverse individuals and other lesbians and bisexual and intersex individuals aged 16 to 24 years). About 35 people attended one or more of the gatherings, which were conducted in “safe spaces” aimed at protecting privacy, trust and confidentiality. All participants were nominated by either a peer or an ally, and each attendee (including organizers) was required to formally commit to not identifying any participants or divulging anything about the discussions.

The young key populations’ dialogue identified a list of priority issues regarding their access to important HIV and broader health and wellness services. These issues included criminalization of same‐sex sexuality, HIV policies that exclude transgender people and a national adolescent sexual and reproductive health strategy that is not responsive to the needs of young key populations. The choice of those three issues was informed by a number of criteria including the impact of the expected change on the daily life of key populations, the timeframe for the implementation of the advocacy plan and the capacity of the group to achieve the results. The outcome of this dialogue informed the design of corresponding policy reform options that were implemented by an advocacy task force composed of elected representatives of young key populations and policy makers appointed at the close of a three‐day workshop.

At that workshop, it was deemed unrealistic by consensus to expect that the sociopolitical context could allow for the successful decriminalization of same‐sex relationships in Zimbabwe. Therefore, the group resolved to focus on the two other top issues: transgender policy inclusion and the review of the adolescent sexual and reproductive health strategy to make it responsive to the needs of young key populations.

The advocacy strategy developed in the first year of the project served as a master plan, and implementation was monitored through quarterly meetings of members of the advocacy task force. Furthermore, youth retreats held annually served as peer‐accountability forums to hear from the local implementing organization about what had been achieved in ensuring inclusion and empowerment of young key populations. Several government‐led meetings were held in 2019 and 2020 to revise the adolescent sexual and reproductive health strategy, and young key population representatives were invited and made significant inputs into the process. The final version of the revised strategy is expected to be released by the end of 2021, and the inclusion of transgender populations as well as recommendation for comprehensive sexuality education for young people will be the indicators of success for the advocacy task team. As a milestone, programmes focusing on transgender populations were included in the 2020‐2022 funding request that was approved by the Global Fund [4].

An important outcome by the end of the third year was the emergence of a young trans‐ and intersex‐led group, called Trans and Intersex Rising in Zimbabwe (TIRZ), previously hosted by the mainstream Gays and Lesbians Association of Zimbabwe (GALZ). TIRZ is comprised of new youth leaders and advocates who are using social media and other approaches to highlight the priorities and needs of young key populations and the importance of the two top issues agreed on during the dialogues. This could not be more timely and relevant in the current context of virtual engagement dictated by the COVID‐19 constraints.

The novel model of participatory dialogue and the leading role of young key population representatives engaging policy makers were used to bring about change in Southern Africa. The case of Zimbabwe teaches us an interesting lesson: with the support of civil society allies, young key population groups can successfully take up the challenge of engagement and visibility in national and regional advocacy processes in a way that has not been witnessed before. This includes addressing matters that traditionally polarize national stakeholders and are often driven by political efforts to win the sympathy of a public influenced by narratives rooted in religious and cultural homophobia. It is worth replicating this model in other regions of sub‐Saharan Africa whilst taking into account the realities of virtual policy engagement resulting from current COVID‐19 restrictions on travel and in‐person meetings.

## COMPETING INTERESTS

The authors have declared no conflict of interest.
